# Five-Year Effectiveness and Adherence Outcomes of SARS-CoV-2 Vaccination in Immunocompromised Patients Since the Launch of the Vaccination Campaign

**DOI:** 10.3390/vaccines14100852

**Published:** 2026-09-28

**Authors:** Adele Sarcone, Maria Rosaria Deodato, Alessandra De Pasquale, Caterina De Sarro, Carmelo G. A. Nobile, Claudia Pileggi

**Affiliations:** 1 Department of Health Sciences, University “Magna Græcia” of Catanzaro, 88100 Catanzaro, Italy; adele.sarcone@studenti.unicz.it (A.S.); mrdeodato83@libero.it (M.R.D.); alessandra.depasquale@studenti.unicz.it (A.D.P.); catedesarro@gmail.com (C.D.S.); 2 Department of Clinical and Experimental Medicine, University “Magna Græcia” of Catanzaro, 88100 Catanzaro, Italy; nobile@unicz.it

**Keywords:** clinical outcomes, immunocompromised individuals, longitudinal vaccine adherence, SARS-CoV-2 immunization, humoral immunity

## Abstract

Background: Immunocompromised patients are at heightened risk for severe COVID-19 and its complications. While vaccination is critical for this population, long-term adherence and the durability of vaccine-induced immunity remain significant public health concerns. This study aimed to evaluate the longitudinal serological response associated with hybrid immunity and to assess temporal trends in SARS-CoV-2 vaccination adherence among immunocompromised patients. Methods: A prospective observational cohort study was conducted on 170 immunocompromised patients followed longitudinally for five years at an Italian Teaching Hospital. Humoral response was assessed by quantifying IgG antibodies against the spike receptor-binding domain (RBD) of SARS-CoV-2 (anti-RBD IgG titers) at multiple time points, from baseline through the first booster dose. Vaccination uptake and reasons for vaccine refusal were monitored throughout the study period. Results: Of the 170 enrolled patients, 159 (94.1%) completed the primary three-dose schedule. Following the initial two-dose regimen, 88% of patients were classified as non- or low-responders (mean anti-RBD IgG: 84.9 BAU/mL). Administration of an additional third dose and subsequent booster markedly enhanced immunogenicity, increasing the mean anti-RBD IgG titer to 3447.5 BAU/mL, with 87.7% of patients classified as high responders post-booster. Despite favorable clinical outcomes, with no COVID-19-related deaths recorded, vaccination uptake demonstrated a significant and continuous decline, dropping from 97% during the pandemic phase to 8.3% during the 2025–2026 campaign. Primary reasons for refusal included doubts regarding vaccine efficacy (55%) and weak indications or contraindications provided by healthcare professionals (22%). Conclusions: Repeated SARS-CoV-2 vaccine doses significantly improve humoral immunity and clinical outcomes in immunocompromised individuals. However, the progressive decline in long-term vaccine uptake, driven largely by efficacy concerns and insufficient provider recommendations, poses a major public health challenge.

## 1. Introduction

Immunization programs have been implemented worldwide to promote vaccination of fragile patients that could lead to an increase in disability and mortality, associated with vaccine preventable disease (VPDs) [1].

Starting in December 2020, SARS-COV2 vaccination should be carried out as a priority in immunocompromised subjects due to the increased risk of severe COVID-19, hospitalization, intensive care unit admission and death [2,3].

In both immunocompetent and immunocompromised individuals, vaccination has been shown to decrease the probability of in-hospital mortality. Thus, even though vaccinated immunocompromised patients still experience a high rate of hospitalization, the procedure represents a critical and advantageous intervention for this population [4,5,6].

Since a SARS-CoV-2 vaccine specifically developed for immunocompromised subjects is not available, it has become essential to adopt strategies aimed at enhancing their immune response. In Italy, additional doses were introduced to complete the primary vaccination series [7,8], and seasonal boosters have been administered starting in October of each year. However, the recommendation by the United States Centers for Disease Control and Prevention (US CDC), which suggests increasing the seasonal vaccine dose to two, spaced six months apart, to ensure adequate protection until the subsequent campaign [9], has not been implemented in the Italian context.

Despite such evidence and the proven safety of the vaccine in fragile subjects, extensively demonstrated by data from the massive participation in the initial vaccination campaign [10], a steady decline in adherence has been observed over time, even among the most vulnerable patients. In the United States, for instance, while over 92% of elderly and vulnerable adults completed the primary vaccination series [11], the adherence rate for the seasonal dose among adults aged 65 and older plummeted to 41.5% in 2023–2024 and further contracted to 32.1% during the 2024–2025 campaign [12].

In Europe, this downward trend was even more pronounced; specifically, seasonal SARS-CoV-2 vaccine coverage among adults aged 60 and older in Italy plummeted from 33.5% during the 2023–2024 season [13] to 10.9% in the 2024–2025 season. This figure aligned with the overall EU/EEA median of 7.4% across reporting countries, before further declining to 6.4% during the 2025–2026 vaccination campaign [14].

Although vaccine-induced immunity wanes over time, it remains crucial in enabling even highly vulnerable individuals to effectively cope with SARS-CoV-2 infection. Furthermore, natural infection induces a protective immune response characterized by antibody lineages that differ from those elicited by vaccination alone [15]. Consequently, the combination of prior infection and vaccination, commonly referred to as hybrid immunity, significantly reduces the risk of subsequent SARS-CoV-2 infection [16]. Given that hybrid immunity provides broader, more robust, and longer-lasting protection across multiple clinical outcomes, vaccination of previously infected individuals continues to represent an important public health and clinical priority [17].

Therefore, in light of these considerations, this study aimed to evaluate, in a real-world cohort of immunocompromised patients, the degree of SARS-CoV-2 seroconversion associated with hybrid immunity resulting from vaccination and natural infection, as well as to assess temporal trends in adherence to SARS-CoV-2 vaccination across the various phases of the COVID-19 pandemic.

## 2. Materials and Methods

### 2.1. Study Design and Setting

A prospective observational cohort study was conducted at the Teaching Hospital of the “Magna Graecia” University of Catanzaro (Southern Italy), as an extension of a previous investigation [18] evaluating BNT162b2 mRNA vaccine in individuals belonging to the priority group for SARS-CoV-2 vaccination. Among the frail subjects enrolled at the COVID-19 vaccination center of the Teaching Hospital starting from 27 December 2020, we identified all immunocompromised patients who were longitudinally followed for five years and three months after the completion of the primary SARS-CoV-2 vaccination cycle. Immunocompromised status included primary or secondary immunodeficiency due to active cancer, solid-organ or hematopoietic transplantation, asplenia, end-stage kidney disease (ESKD), or ongoing treatment with immunosuppressive or immunomodulatory medications.

After enrollment, subjects signed informed consent and underwent venous blood sampling for serological analysis. Four blood samples were collected for each participant to detect antibodies against SARS-CoV-2: at the time of the first vaccine dose (baseline—T0), after 21 days, at the time of the second vaccine dose (T1), after 30–180 days of the second dose (T2), 30 days after the administration of the additional dose (hereinafter referred as third dose) (T3) indicated for the immunocompromised subjects and, 30 days after the first booster dose (T4). Serological follow-up was performed from baseline (T0) through T4, corresponding to 30 days after the first booster dose. Thereafter, patients continued to be followed longitudinally through the subsequent seasonal SARS-CoV-2 vaccination campaigns (2022–2023 to 2025–2026). During this follow-up period, vaccine effectiveness was evaluated through the assessment of clinical outcomes, including SARS-CoV-2 infection and related adverse events, such as hospitalization and death, without the collection of additional serological samples.

The Abbott SARS-CoV-2 IgG II Quant assay and Architect i1000RS instrument (Abbott Diagnostic, Abbott Park, IL, USA). The assay is a chemiluminescent microparticle immunoassay (CMIA) that was used to quantify IgG antibodies against the spike receptor-binding domain (RBD) of SARS-CoV-2. In line with the World Health Organization (WHO) standards, the initial value expressed in arbitrary units (AU)/mL was converted to binding antibody units (BAU)/mL using the conversion factor provided by Abbott, whereby 1 BAU/mL corresponds to 0.142 Abbott AU/mL [19]. Cut-off values were defined according to Dekervel et al. [20] as non-responders with levels ≤7.1 BAU/mL, low responders with levels between 7.2 and 142 BAU/mL, and high responders with levels ≥142.1 BAU/mL.

For each patient, at the time of the first vaccine dose, a medical record was completed to collect information about demographic characteristics and clinical data, focusing attention on the cause of the immune impairment (e.g., type and date of transplantation, primary kidney disease, type and duration of dialysis, type of cancer, immunosuppressant treatments, comorbidities, etc.). The age-adjusted Charlson co-morbidity index was calculated as a marker of the individual burden of disease.

Patients were followed longitudinally through scheduled telephone contacts: once per month during the first year after the start of the SARS-CoV-2 vaccination campaign, and twice per year thereafter, in correspondence with the seasonal vaccination cycles, up to the 2025–2026 vaccination campaign. At each follow-up contact, additional information was collected to update patients’ baseline clinical conditions and to record any SARS-CoV-2 infections. In the event of a reported infection, details were obtained regarding the severity of clinical manifestations, treatments received, and any need for hospitalization. Patients were also asked whether they had received the recommended seasonal influenza and pneumococcal vaccinations.

### 2.2. Statistical Analysis

Statistical analysis was performed using STATA software program, version 18 (Stata Corporation, College Station, TX, USA). Data was summarized using frequencies and percentages for categorical data and means and standard deviations (SD) for continuous data. Chi-square analyses or Fisher’s exact tests were used to compare categorical variables and ANOVA was used for continuous variables.

The first outcome of interest was the anti-S IgG titer measured 30 days after the third dose of SARS-CoV-2 vaccination (T3), expressed in BAU/mL, reflecting the immune response conferred by the combination of vaccination and natural infection (hybrid immunity). The anti-RBD IgG titer was categorized into three levels according to the cut-off values proposed by Dekervel et al. [20].

A multinomial logistic regression model was developed to assess the potential role of the following independent variables: age, sex, age-adjusted Charlson Comorbidity Index, number of chronic diseases, underlying cause of immune impairment, total number of SARS-CoV-2 vaccine doses received, and number of SARS-CoV-2 infections experienced.

The second outcome aimed to evaluate whether immunocompromised patients modified their adherence to SARS-CoV-2 vaccination during the evolving phases of the pandemic. To assess changes in adherence over time, the proportions of vaccinated individuals across pandemic phases were compared using a chi-square test. This study is reported according to the STROBE guidelines for observational studies (Supplementary Materials—Checklist S1) [21].

### 2.3. Ethics

The study protocol was ratified by the Approval from the Territorial Ethics Committee of the Calabria Region (ID N. 183/20 May 2021).

## 3. Results

From 27 December 2020 to 31 March 2026, 427 frail patients were enrolled at the COVID-19 vaccination center, of whom 170 met the criteria for immunocompromised status and were included in the longitudinal cohort. One patient declined vaccination and subsequently died from COVID-19-related complications. Among the remaining 169 patients, five refused the additional third dose and five died from underlying diseases during the primary SARS-CoV-2 vaccination course. Consequently, 159 patients (94.1%) completed the primary three-dose vaccination schedule. Regarding the first booster dose, among the 158 eligible patients (one additional patient died from an underlying disease), 28 declined vaccination (Supplementary Materials Figure S1).

All patients were followed longitudinally until death or for a maximum of five years. The main characteristics of the study population are reported in Table 1. The median age of the cohort was 65 years (IQR 58–72), and 26% were females. Approximately 60% of patients had three or more non-communicable diseases (NCDs), with cardiovascular conditions (72.2%), dysmetabolic disorders (23.1%), and diabetes mellitus (20.1%) being the most prevalent. The leading cause of immunosuppression was organ transplantation (62.7%), mainly liver (71.7%) and kidney (25.5%) transplantation. The mean duration of immunosuppression was 8.6 years (IQR 3–11).

With respect to SARS-CoV-2 infections (Table 1), 3% of patients had detectable anti-S IgG at the time of the first vaccine dose, indicating a previous infection. The highest infection rate was observed after the start of the vaccination course, particularly following the booster dose (48.9). Among patients who reported SARS-CoV-2 infection, only 11.1% experienced severe symptoms, and hospital admission to the Intensive Care Unit (ICU) was required for just one patient. None of the subjects included in the study died as a consequence of SARS-CoV-2 infection or related complications.

The distribution of immunocompromised patients according to anti-RBD IgG antibody levels and the number of SARS-CoV-2 vaccine doses received is shown in Figure 1.

After completion of the primary two-dose SARS-CoV-2 vaccination schedule, 88% of patients were classified as non- or low-responders, with a mean anti-RBD IgG titer of 84.9 BAU/mL (Figure 2a). Administration of the additional third vaccine dose significantly enhanced the serological response, reducing the proportion of non- or low-responders to 29.4% and significantly increasing mean anti-RBD IgG titers compared with those measured after the second dose (paired *t*-test = −8.435, df = 124, *p* < 0.0001) (Figure 2a). Administration of the booster dose further strengthened the humoral immune response, increasing the mean anti-RBD IgG titer to 3447.5 BAU/mL and reducing the proportion of non- or low-responders to 12.3%, while high responders accounted for 87.7% of the evaluated patients (Figure 2b).

To further investigate the impact of prior SARS-CoV-2 infection on the humoral immune response, anti-RBD IgG levels following the first booster dose (T4) were compared between patients with vaccine-induced immunity only and those with hybrid immunity. The comparison revealed no statistically significant difference in mean anti-RBD IgG titers between the two groups (*t*-test = 0.447, df = 134, *p* = 0.655) (Figure 3).

Univariate and multivariate analyses did not show any significant association between anti-RBD IgG titer measured at T3 and the independent variables tested (Supplementary Materials Tables S1 and S2).

Finally, regarding vaccination uptake across the study period (Figure 4), a constant and significant decline in adherence among immunocompromised patients after completion of the primary three-dose cycle was observed. In the most recent seasonal dose (2025–2026), adherence dropped to 8.3%, compared with the 97% adherence recorded during the SARS-CoV-2 pandemic period (χ^2^ = 234.1, 1 df, *p* < 0.0001). Among the principal reasons for refusing SARS-CoV-2 vaccination, patients most frequently reported doubts about vaccine efficacy in preventing infection (55%), followed by weak indication or contraindication provided by their physician (22%), fear of adverse effects (11.5%), and the perception of being at low risk of infection (8%).

Influenza vaccination uptake also declined during the last season compared with the levels observed during the early phase of the SARS-CoV-2 pandemic, although the decrease was less pronounced than that observed for SARS-CoV-2 vaccination. In contrast, no significant changes in pneumococcal vaccination uptake were detected throughout the follow-up period. Further analyses focusing on influenza and pneumococcal vaccination behaviors are currently ongoing and will be reported separately.

## 4. Discussion

In this longitudinal cohort of immunocompromised patients followed for up to five years, SARS-CoV-2 vaccination was associated with a progressive and substantial enhancement of humoral immunity. Although the initial two-dose vaccination schedule elicited a limited serological response, with nearly 90% of participants classified as non- or low responders, administration of the additional third dose and subsequent booster markedly increased anti-RBD IgG titers and a significant reduction in the proportion of poor responders. These findings support the pivotal role of extended vaccination schedules in overcoming, at least partially, the impaired vaccine responsiveness traditionally observed in immunocompromised populations.

The limited response observed after the first two doses is consistent with our previous findings in frail patients [18] and with studies involving immunosuppressed populations, including solid organ transplant recipients [22,23]. Underlying diseases and immunosuppressive therapies may impair both B- and T-cell responses, resulting in reduced seroconversion and antibody production. Furthermore, several studies have shown that administration of additional vaccine doses can partially overcome impaired immune responsiveness, leading to a significant increase in antibody production and improving overall vaccine immunogenicity [24,25]. Consistent with these observations, one of the key findings of the present study was the marked benefit associated with administration of a third vaccine dose. Following this additional dose, the prevalence of non- or low-responders decreased substantially, accompanied by a significant increase in anti-RBD IgG levels. These results reinforce the concept that immunocompromised patients require vaccination schedules specifically tailored to their immune status and support current recommendations advocating enhanced primary immunization regimens in vulnerable individuals. Importantly, the beneficial effect of repeated immunization was not limited to the third dose. The subsequent booster further enhanced the humoral response, with nearly 90% of evaluated patients being classified as high responders. Consistent with this observation, anti-RBD IgG levels after booster vaccination were comparable between patients with vaccine-induced immunity alone and those with hybrid immunity, suggesting that repeated vaccine doses may substantially contribute to the development of a robust humoral response in this population.

The relatively high incidence of SARS-CoV-2 infection observed in our cohort during follow-up, particularly after booster administration, is consistent with reports from several countries during the period of Omicron predominance. The emergence of Omicron and its sublineages was associated with a marked increase in breakthrough infections among vaccinated individuals due to their enhanced ability to evade vaccine-induced immunity. Nevertheless, vaccination continued to provide substantial protection against severe disease, hospitalization, and death, especially in high-risk populations [26,27]. Our findings are in line with these observations. Although impaired vaccine responsiveness persisted in a subset of patients, clinical outcomes remained generally favorable throughout follow-up. Severe COVID-19 was uncommon; only one patient required ICU admission, and no COVID-19-related deaths were recorded among the study participants. These results suggest that, even when humoral responses are suboptimal, vaccination may still confer clinically meaningful protection against severe outcomes, potentially through residual antibody activity and/or vaccine-induced cellular immunity [28,29].

A major finding of our study was the marked decline in vaccination uptake over time. Adherence was very high during the initial pandemic vaccination campaign and the primary three-dose schedule but progressively decreased during subsequent seasonal campaigns, reaching only 8.3% in 2025–2026. This trend mirrors the broader decline in COVID-19 booster uptake observed in Italy and internationally [30,31] and therefore should not be considered specific to immunocompromised patients.

The reasons for vaccine refusal provide further insight into this phenomenon. Doubts about vaccine effectiveness were the most frequently reported concern, followed by weak recommendations or contraindications from healthcare providers, fear of adverse effects, and a perceived low risk of infection. Notably, 22% of patients cited physician-related recommendations as a barrier to vaccination, highlighting the influence of healthcare professionals on vaccine uptake. This finding is consistent with evidence showing that frail patients often rely on their General Practitioner for vaccination advice [32], while concerns about vaccine safety have also been reported among healthcare workers [33]. These findings underscore the importance of consistent, evidence-based counseling to improve vaccine uptake in immunocompromised populations [1,34].

This study has several limitations. First, it was conducted in a single vaccination center, potentially limiting the generalizability of the findings. Second, the decreasing number of participants receiving subsequent vaccine doses may have introduced selection bias in the assessment of later immunogenicity. Third, the heterogeneous nature of the immunocompromised population may have obscured differences among specific clinical subgroups. Finally, it should be noted that the present study did not directly assess functional neutralizing activity; therefore, the observed increase in binding antibody levels should not be interpreted as a direct measure of viral neutralization capacity.

## 5. Conclusions

In conclusion, despite these limitations, this study provides real-world evidence that repeated SARS-CoV-2 vaccine doses substantially enhanced serological responses in immunocompromised patients and were associated with favorable clinical outcomes, even amid ongoing viral circulation. Nevertheless, the marked decline in vaccination uptake represents a major public health concern that may compromise long-term protection in this vulnerable population. Future strategies should therefore aim not only to optimize vaccine-induced immunity but also to address vaccine hesitancy through patient-centered and healthcare provider–driven interventions.

## Figures and Tables

**Figure 1:**
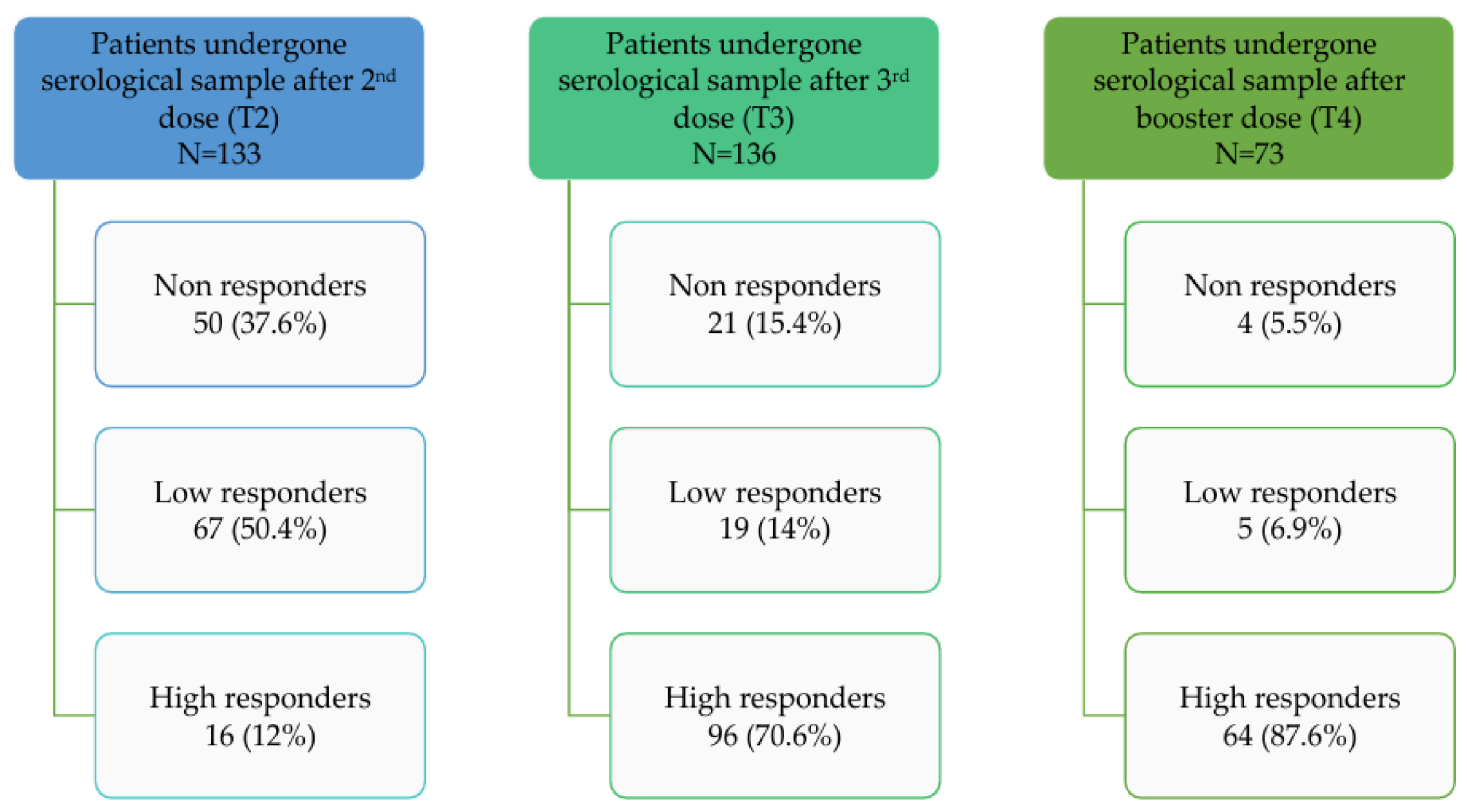
Humoral immunity profile of immunocompromised subjects categorized by anti-RBD IgG titers across sequential SARS-CoV-2 immunization stages.

**Figure 2:**
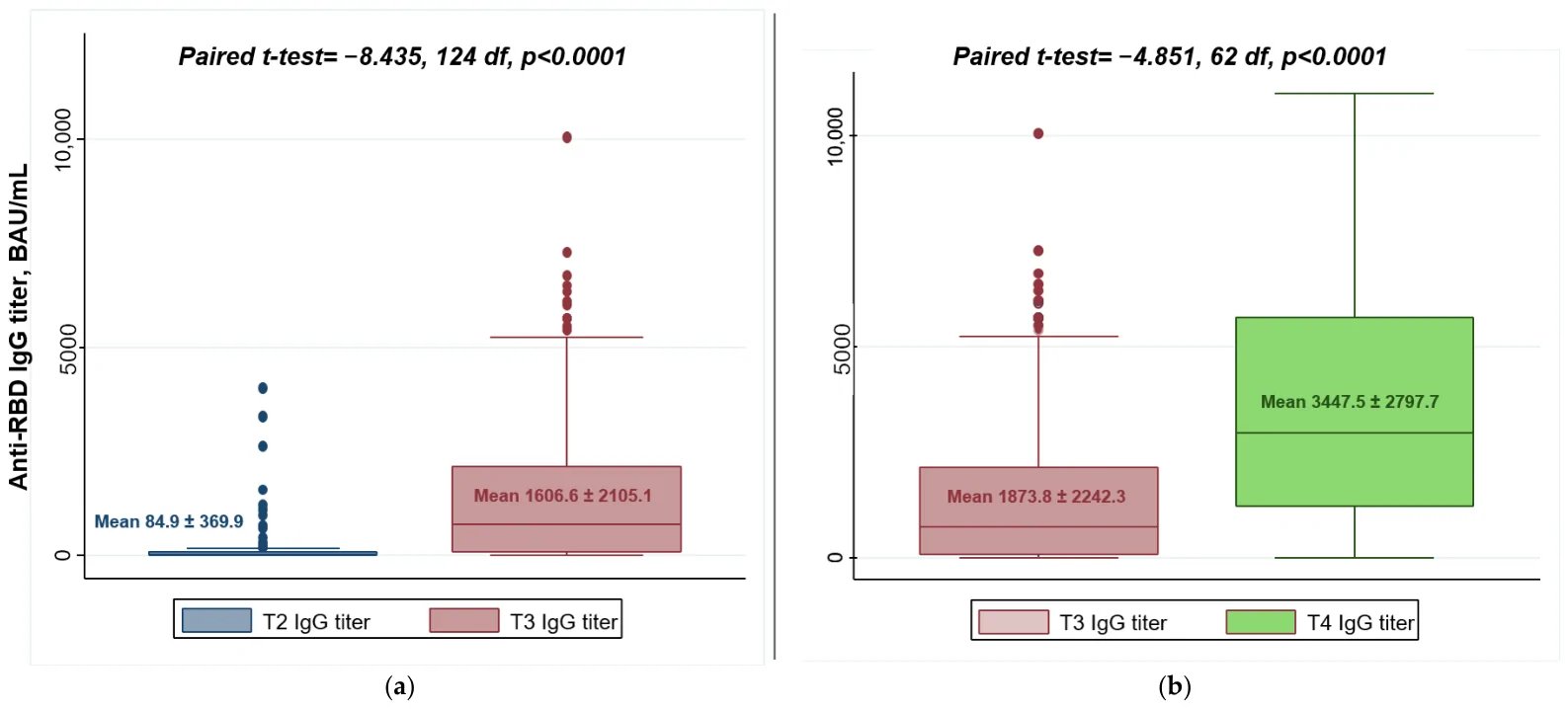
Box-plot distribution of humoral response levels relative to immunization frequency: (**a**) comparative analysis of anti-RBD IgG titers post-primary series (T2) versus post third dose (T3); (**b**) comparative analysis of anti-RBD IgG titers post third dose (T3) versus the first booster (T4).

**Figure 3:**
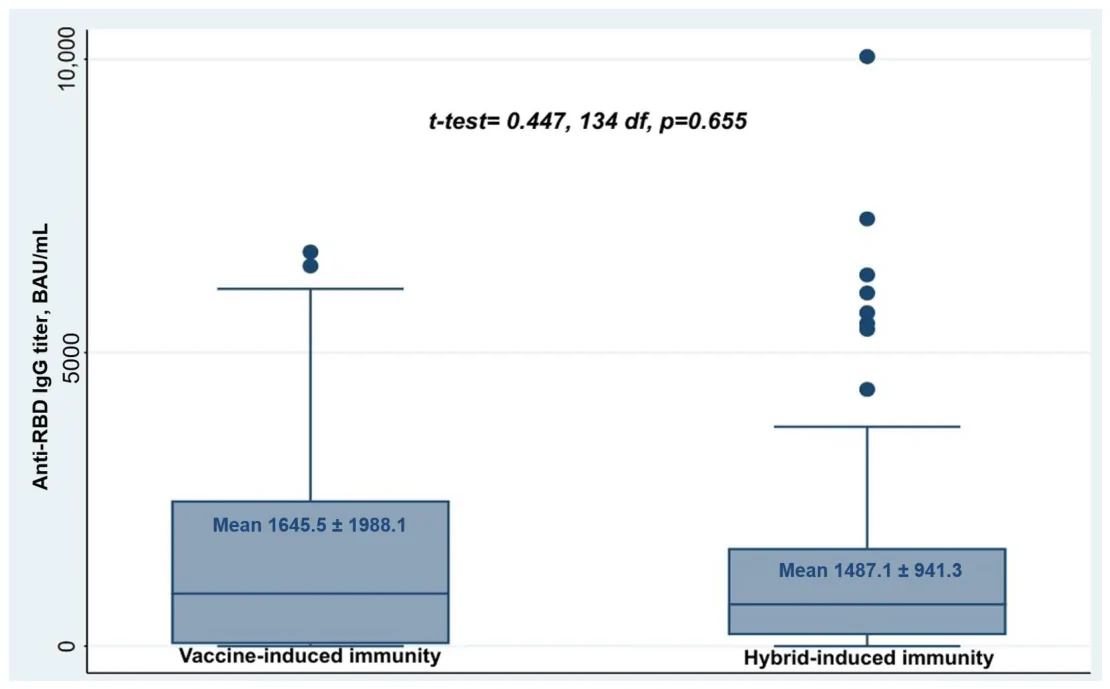
Box plot showing the distribution of anti-RBD IgG antibody levels following the first booster dose (T4) in patients with vaccine-induced immunity and hybrid immunity.

**Figure 4:**
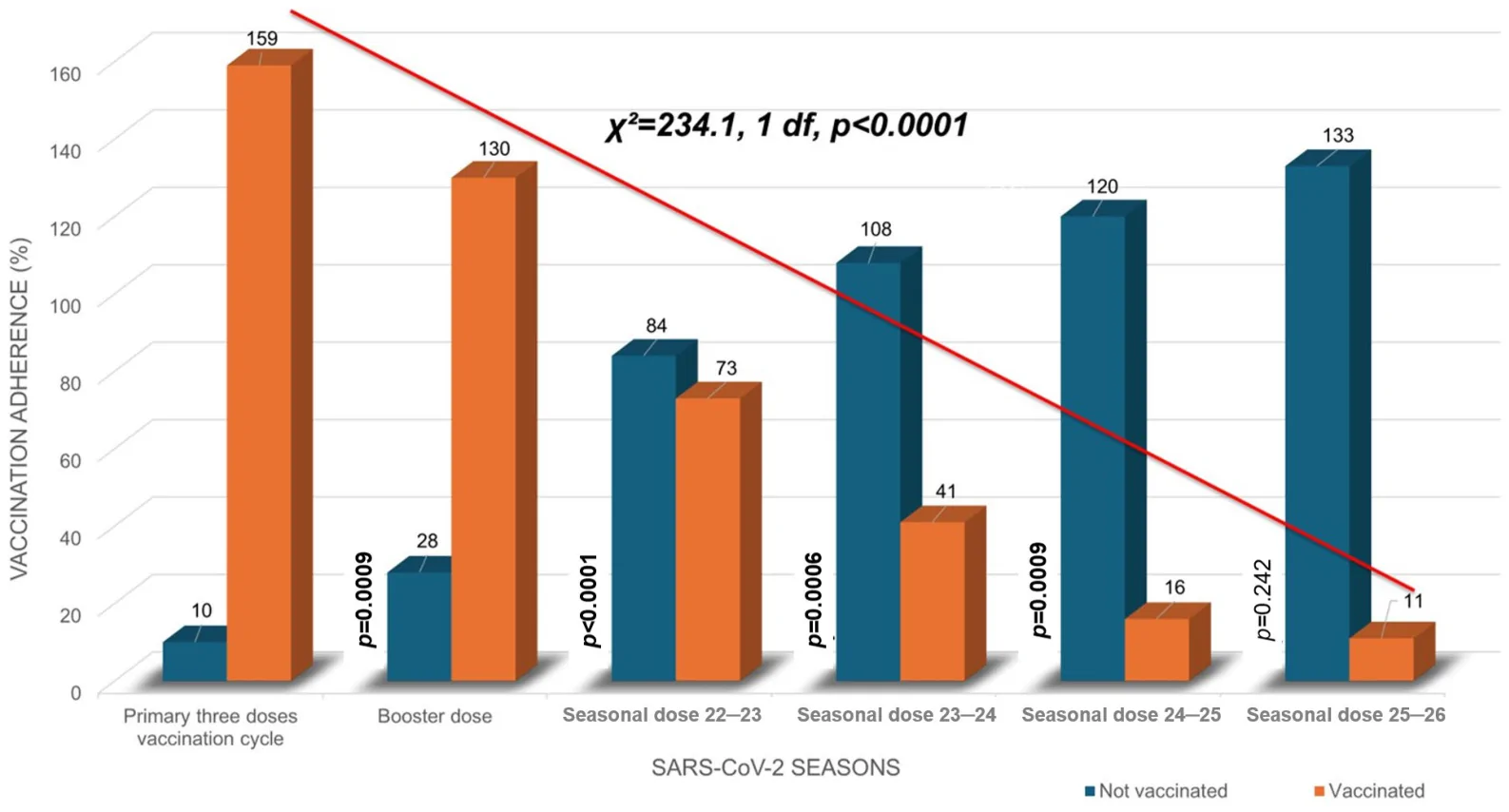
Comparative evaluation of longitudinal SARS-CoV-2 immunization uptake from the 2020–2021 period through the 2025–2026 vaccination campaign.

**Table 1:** Demographics and clinical characteristics of the study population.

Characteristic		N (%)
Sex		
Female		44 (26%)
Age, years Mean ± SD; Interquartile Range (IQR)		65 ± 11.1; 14 (58–72)
≤45		9 (5.3%)
46–64		64 (37.8%)
≥65		96 (56.8%)
Age-adjusted Charlson comorbidity index (CCIa) Mean ± SD		6.4 ± 2.12
Total number chronic disease		
≥3		102 (60.4%)
Coexisting conditions ^a^		
Cardiovascular disease		122 (72.2%)
Dysmetabolic disease		39 (23.1%)
Diabetes mellitus		34 (20.1%)
Gastrointestinal diseases		34 (20.1%)
Respiratory disease		21 (12.4%)
Cancer		15 (8.9%)
Neurological disease		6 (3.5%)
Cause of immune impairments ^a^		
Organ Transplant		106 (62.7%)
Liver		76 (71.7%)
Kidney		27 (25.5%)
Other combined transplants ^b^		3 (2.8%)
End stage of kidney disease (ESKD) ^c^		51 (30.2%)
Other causes of immune impairments ^d^		12 (7.1%)
Duration of immune impairment, years		
Mean ± SD; Interquartile Range (IQR) (146)		8.6 ± 8.7; 13 (3–11)
≤1		8 (4.7%)
2–5		67 (39.6%)
6–9		29 (17.2%)
≥10		42 (24.9%)
Do not remember		23 (13.6%)
Total number of vaccine doses received		
Primary three-dose vaccination series		159 (96.9%)
Booster dose (158)		130 (82.3%)
Seasonal dose 22–23 (157)		73 (46.5%)
Seasonal dose 23–24 (149)		41 (27.5%)
Seasonal dose 24–25 (136)		16 (11.8%)
Seasonal dose 25–26 (133)		11 (8.3%)
SARS-CoV-2 infection		
No/ineligible/not remember		78 (46.1%)
Yes, before the vaccination		5 (3%)
Yes, after the start of the vaccination cycle:		86 (50.9%)
	primary vaccination series	23 (26.7%)
	booster dose	42 (48.9%)
	seasonal dose	21 (24.4%)

Values in brackets represent the count of eligible patients whenever this figure deviates from the total cohort of 169; SD: Standard Deviation. ^a^ Percentages do not add up to 100 due to multiple responses. ^b^ Other combined transplants include: 2 kidney-liver and 1 kidney-pancreas. ^c^ Six patients were transplant recipients and were therefore assigned to the category with the highest level of immunodeficiency. ^d^ Other causes of immune impairment include: 1 lymphoma, 1 breast cancer, 2 adenocarcinoma of the large intestine, 1 multiple sclerosis, 1 psoriatic arthritis, 3 cardiovascular diseases, 1 down syndrome and 2 dysmetabolic disorders.

## Data Availability

All data generated or analysed during the study are included in this published article. The dataset used during the current study is available from the corresponding author on reasonable request.

## Supplementary Materials

The following supporting information can be downloaded at: https://www.mdpi.com/article/10.3390/vaccines14100852/s1, Figure S1: STROBE flow diagram of study participants; Table S1: Univariate analysis of factors associated with anti-RBD IgG titer levels 30 days after the additional third SARS-CoV-2 vaccine dose (T3); Table S2: Result of the multinomial regression analysis between anti-RBD IgG titer measured at T3 and the independent variables tested (reference category: high responder); Checklist S1: STROBE Statement—checklist of items that should be included in reports of observational studies.

## Abbreviations

The following abbreviations are used in this manuscript:

AU	Arbitrary Units
BAU	Binding Antibody Units
CDC	Centers for Disease Control and Prevention
CI	Confidence Interval
ESKD	End-Stage Kidney Disease
ICU	Intensive Unit Care
IQR	Interquartile Range
NCD	Non-Communicable Disease
RBD	Receptor Binding Domain
SD	Standard Deviation
VPD	Vaccine-Preventable Disease
WHO	World Health Organization
