# Supplementary material for: Five-Year Effectiveness and Adherence Outcomes of SARS-CoV-2 Vaccination in Immunocompromised Patients Since the Launch of the Vaccination Campaign
Source: Vaccines (Basel). 2026 Sep 28;14(10):852. doi: 10.3390/vaccines14100852 (PMC13709813; doi:10.3390/vaccines14100852)
Supplement: Supplementary file 1 [file vaccines-14-00852-s001.zip › Figure S1.pdf]

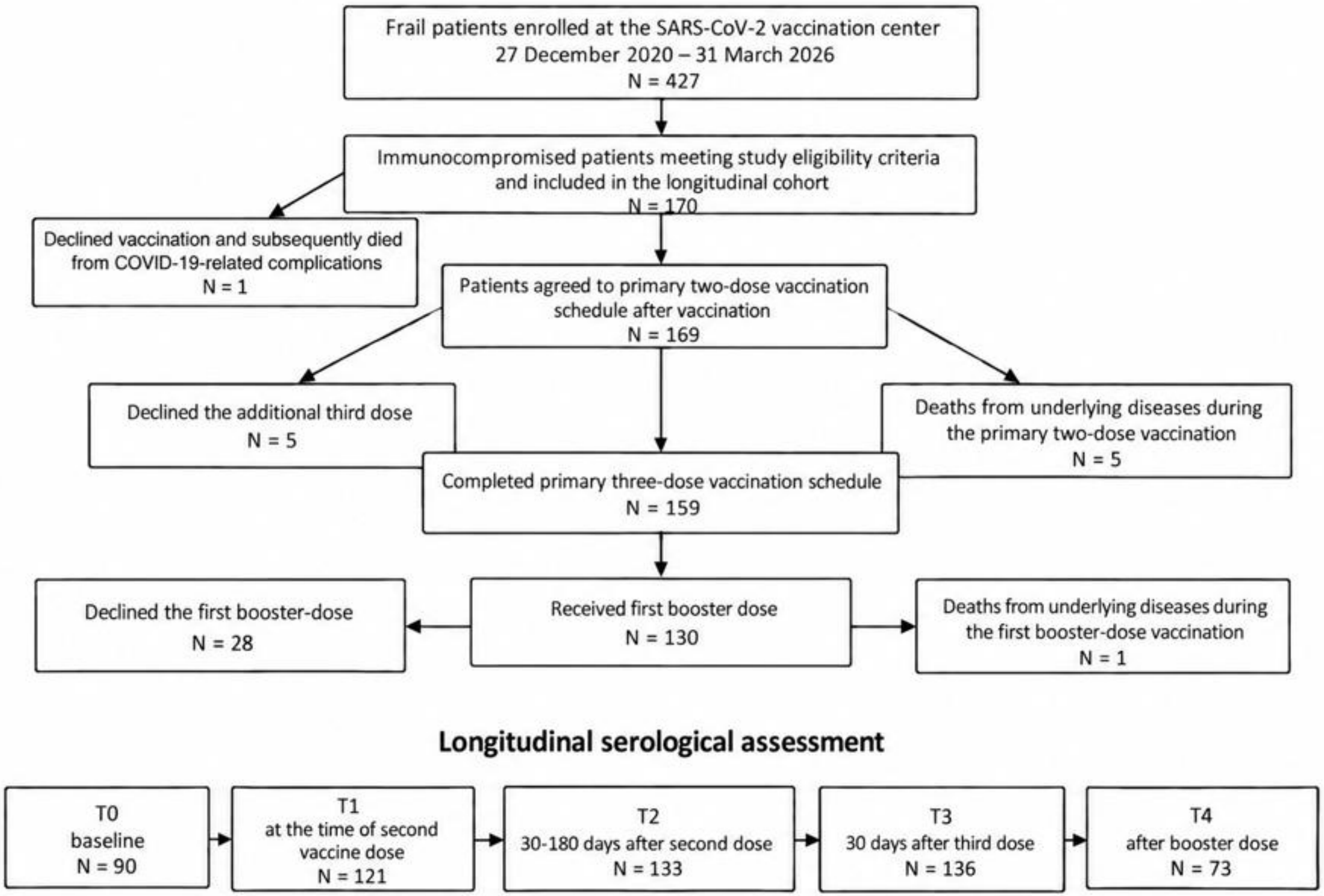

Figure S1: STROBE flow diagram of study participants
