# Supplementary material for: Five-Year Effectiveness and Adherence Outcomes of SARS-CoV-2 Vaccination in Immunocompromised Patients Since the Launch of the Vaccination Campaign
Source: Vaccines (Basel). 2026 Sep 28;14(10):852. doi: 10.3390/vaccines14100852 (PMC13709813; doi:10.3390/vaccines14100852)
Supplement: Supplementary file 1 [file vaccines-14-00852-s001.zip › Table S1.pdf]

Table S1. Univariate analysis of factors associated with anti-RBD IgG titer levels 30 days after the additional third SARS-CoV-2 vaccine dose (T3)

Characteristic	Total		Non responder		Low responder		High responder	
	N	%	N	%	N	%	N	%
	(136)	100	(21)	15.4	(19)	14	(96)	70.6
Socio-demographic profile								
**Sex**								
Male	99	72.8	14	14.1	14	14.1	71	71.8
Female	37	27.2	7	18.9	5	13.5	25	67.6
					$\chi^2 = 0.471$, 2df, p=0.790			
**Age, years**								
≤ 45	7	5.2	4	57.1	1	14.3	2	28.6
46-64	52	38.2	10	19.2	10	19.2	32	61.6
≥ 65	77	56.6	7	9	8	10.4	62	80.6
					Fisher exact p=0.005			
Medical conditions								
**Age-adjusted Charlson comorbidity index (CCIa)**	6.4±2.1		5.5±2.5		6.4±2.2		6.6±2	
					F=257, p=0.081			
**Total number chronic disease**								
≤ 2	55	40.4	11	20	7	12.7	37	67.3
≥ 3	81	59.6	10	12.4	12	14.8	59	72.8
**Coexisting conditions**								
**Cardiovascular disease**								
No	33	24.2	8	24.2	3	9	22	66.8
Yes	103	75.8	13	12.6	16	15.5	74	71.9

Characteristic	Total		Non responder		Low responder		High responder	
	N	%	N	%	N	%	N	%
	(136)	100	(21)	15.4	(19)	14	(96)	70.6
					Fisher exact *p*=0.241			
**Dysmetabolic disease**								
No	105	77.2	15	14.3	14	13.3	76	72.4
Yes	31	22.8	6	19.4	5	16.1	20	64.5
					χ^2 = 0.742, 2df, *p*=0.690			
**Diabetes mellitus**								
No	107	78.7	18	16.8	13	12.2	76	71
Yes	29	21.3	3	10.3	6	20.7	20	69
					Fisher exact *p*=0.436			
**Gastrointestinal disease**								
No	107	78.7	20	18.7	15	14	72	67.3
Yes	29	21.3	1	3.4	4	13.8	24	82.8
					Fisher exact *p*=0.117			
**Respiratory disease**								
No	119	87.5	19	16	15	12.6	85	71.4
Yes	17	12.5	2	11.8	4	23.5	11	64.7
					Fisher exact *p*=0.467			
**Cancer**								
No	125	91.9	17	13.6	19	15.2	89	71.2
Yes	11	8.1	4	36.4	0	0	7	63.6
					Fisher exact *p*=0.077			
**Neurologic disease**								
No	131	96.3	20	15.3	19	14.5	92	70.2
Yes	5	3.7	1	20	0	0	4	80
					Fisher exact *p*=1.000			
**Cause of immune**								

Characteristic	Total		Non responder		Low responder		High responder	
	N	%	N	%	N	%	N	%
	(136)	100	(21)	15.4	(19)	14	(96)	70.6
**impairments**								
Organ Transplant	92	67.6	17	18.5	14	15.2	61	66.3
End stage of kidney disease (ESKD)	34	25	2	5.9	5	14.7	27	79.4
Other cause of immune impairments	10	7.4	2	20	0	0	8	80
					Fisher exact *p*=0.282			
**Duration of immune impairment, years**								
≤ 1	3	2.2	1	33.3	0	0	2	66.7
2-5	56	41.2	9	16.1	10	17.9	37	66
6-9	26	19.1	4	15.4	2	7.7	20	76.9
≥ 10	36	26.5	3	8.3	5	13.9	28	77.8
Do not remember	15	11	4	26.7	2	13.3	9	60
					Fisher exact *p*=0.645			
**SARS-CoV-2 infection**								
No/Yes after additional dose	114	83.8	16	14	17	14.9	81	71.1
Yes, before additional dose	22	16.2	5	22.7	2	9.1	15	68.2
