# Supplementary material for: Five-Year Effectiveness and Adherence Outcomes of SARS-CoV-2 Vaccination in Immunocompromised Patients Since the Launch of the Vaccination Campaign
Source: Vaccines (Basel). 2026 Sep 28;14(10):852. doi: 10.3390/vaccines14100852 (PMC13709813; doi:10.3390/vaccines14100852)
Supplement: Supplementary file 1 [file vaccines-14-00852-s001.zip › Table S2.pdf]

Table S2. Result of the multinomial regression analysis between anti-RBD IgG titer measured at T3 and the independent variables tested (reference category: high responder)

Log Likelihood = -84.15 ; χ^2=0.277; No. of observation= 117

Outcome: anti-RBD IgG titer 30 days after the administration of the additional third vaccine dose

	**Non responder**		**Low responder**	
	**RRR (95% CI)**	***p* value**	**RRR (95% CI)**	***p* value**
Sex (male as reference)	0.72 (0.17-2.99)	0.651	1.12 (0.27-4.57)	0.875
Age, continuous	0.94 (0.87-1.01)	0.107	0.97 (0.90-1.04)	0.346
Age - adjusted Charlson comorbidity index (CCIa), continuous	1.04 (0.67-1.62)	0.857	0.99 (0.67-1.46)	0.956
Duration of immune impairment, continuous	0.96 (0.89-1.04)	0.355	0.94 (0.86-1.03)	0.228
Total number chronic disease ≤ 2 disease as references)	0.33 (0.09-1.20)	0.093	1.37 (0.37-5.15)	0.637
Cause of immune impairments (Organ transplant as reference)	0.77 (0.19-3.07)	0.715	0.40 (0.09-1.77)	0.225
SARS-CoV-2 infection (No/Yes after booster dose as reference)	1.75 (0.44-6.94)	0.426	0.69 (0.13-3.71)	0.662
